# Microencapsulated Red Powders from Cornflower Extract—Spectral (FT-IR and FT-Raman) and Antioxidant Characteristics

**DOI:** 10.3390/molecules27103094

**Published:** 2022-05-11

**Authors:** Renata Różyło, Monika Szymańska-Chargot, Artur Zdunek, Urszula Gawlik-Dziki, Dariusz Dziki

**Affiliations:** 1 Department of Food Engineering and Machines, University of Life Sciences in Lublin, Głęboka Street 28, 20-612 Lublin, Poland; 2 Institute of Agrophysics, Polish Academy of Sciences, Doświadczalna Street 4, 20-290 Lublin, Poland; a.zdunek@ipan.lublin.pl; 3 Department of Biochemistry and Food Chemistry, University of Life Sciences in Lublin, Skromna Street 8, 20-704 Lublin, Poland; urszula.gawlik@up.lublin.pl; 4 Department of Thermal Technology and Food Process Engineering, University of Life Sciences in Lublin, Głęboka Street 31, 20-612 Lublin, Poland; dariusz.dziki@up.lublin.pl

**Keywords:** cornflower petal, food colorant, microencapsulation, freeze-drying, spectral, FT-IR, FT-Raman, structure, antioxidant

## Abstract

Although the health benefits of cornflower extracts are known, their application in food production has not been widely investigated. This study assessed microencapsulated red powders (RP) prepared from the aqueous extract of blue cornflower petals. Microencapsulation was performed by freeze-drying using various stabilizers, such as maltodextrin, guar gum, and lecithin. The microencapsulated RP were characterized by spectral (FT-IR and FT-Raman), mineral, structural, and antioxidant analyses. The FT-IR and FT-Raman band related to guar gum, lecithin, and maltodextrin dominated over the band characteristic of anthocyanins present in the cornflower petal powders. The main difference observed in the FT-Raman spectra was attributed to a shift of bands which is reflection of appearance of flavium cation forms of anthocyanins. The microencapsulated RP had total phenolic content of 21.6–23.4 mg GAE/g DW and total flavonoid content of 5.0–5.23 mg QE/g. The ABTS radical scavenging activity of the tested powders ranged from 13.8 to 20.2 EC_50_ mg DW/mL. The reducing antioxidant power (RED) of the powders was estimated at between 31.0 and 38.7 EC_50_ mg DW/mL, and OH^•^ scavenging activity ranged from 1.9 to 2.6 EC_50_ mg DW/mL. Microencapsulated cornflower RP can be valuable additives to food such as sweets, jellies, puddings, drinks, or dietary supplements.

## 1. Introduction

Flower petals have multicolored pigments, which attract manufacturers for the production of natural food colorants [1,2,3,4]. Anthocyanins are pigments found in fruits, flowers, and other plant structures [5], and provide a red or blue color [6,7]. Previous studies have shown that flowers that have an intense red or blue color have a high content of these pigments [8,9]. Anthocyanins are colored, water-soluble, and belong to the phenolic group. These compounds exist in glycosylated forms [10] Anthocyanins consist of an anthocyanidin (aglycone) attached to sugar moieties. Studies have shown that anthocyanins have a beneficial effect on health, by affecting the cellular antioxidant state and inflammation [5]. Anthocyanins have also been shown to modulate the composition of the gut microbiome and play overlapping roles in the prevention and treatment of cardiovascular diseases, cancer, and neurodegenerative disorders [11].

Colored anthocyanin pigments have traditionally been used as natural food coloring agents. Their color and stability are influenced by pH, light, and temperature, and depend on structure [10]. Anthocyanins undergo color changes depending on the pH of the solvent [7,12,13,14,15]. At a low acidic pH, these compounds give a red color and are more stable, while at a higher pH, they give a blue color [16].

Cornflower with blue petals has blue pigments [17,18]. The flowers contain protocyanin pigments [19], which, when combined with iron and aluminum, form dark blue complexes. Researchers have shown that the blue color comes from a complex of anthocyanins and flavones with metal ions such as iron, magnesium, and calcium [20,21,22]. A study also identified four cyanide derivatives in cornflower petals [23]. In addition to these compounds, cornflower petals contain tocopherols, organic acids, and apigenin derivatives.

Reports indicate that supplementation of diet with edible flowers as a source of antioxidants may have a beneficial effect on human health. Flowers including roses, French marigold, lavender, heather, elderflower, horned pansy, and cornflower have been shown to act as chemopreventive agents. It has also been emphasized that flowers may protect human DNA from oxidative damage [24].

Despite the proven health benefits, cornflower extracts have not been widely studied in food production. Thus far, only attempts have been made to make use of hydrophilic cornflower extracts as an alternative for anthocyanins in yogurt [25]. The blue pigment of cornflower petals was studied in our previous work [26]. The spectral properties and physiological functions of plant pigments are currently of interest to scientists all around the world [27].

In this study, the red pigment was obtained from cornflower petals and microencapsulated by lyophilization by adding stabilizers.

The scope of the study was the characterization of the color, mineral content, antioxidant properties, microstructure, and spectral properties of microencapsulated red powders (RP) obtained from cornflower extracts. Our study is the first to analyze the molecular characteristics of such products.

## 2. Results and Discussion

### 2.1. Color Values

The color parameters measured in dried cornflower petals (C), red extract (R), and microencapsulated powders (R2G, R4G, R2L) are presented in Table 1. Dried cornflower petals (C) were blue in color, and their L*, a*, b*, C*, and ho values were 32.4, 4.2, −7.9, 8.9, and 297.8, respectively. The red extract (R) obtained from the blue aqueous extract after pH reduction from 4.7 (blue) to 2.2 (red) was deep red in color, and its L*, a*, b*, C*, and h^o^ values were 23.9, 3.8, −1.8, 4.21, and 328, respectively. The structural transformations that the red extract undergoes in aqueous solutions are mostly related to pH changes [12,13,14,28]. A previous study showed that rose anthocyanins serve as a good source of functional ingredients and color in an acidic environment [29].

The powders obtained from the microencapsulation of the extract by freeze-drying were characterized by a different color than the extract, but eventually pink-red powders were obtained. Lightness L* and a* values significantly increased during microencapsulation (Table 1). The darkest RP were obtained when the stabilizer mixture had 4% of guar gum and 6% maltodextrin (R4G) (L* = 59.3; a* = 30.2; b* = −2.7; C* = 30.3; h^o^ = 355.2). On the other hand, lighter powders were obtained with the addition of lecithin (R2L) (L* = 64.8; a* = 26.8; b* = −1.9; C* = 26.9; h^o^ = 355.4). A similar tendency was observed in the case of blue powder obtained in the previous study [26]. Comparing blue powder and RP, the red ones had a lighter shade but their brightness did not significantly differ from that of the blue lecithin sample. As mentioned above, in the colored powders obtained after the microencapsulation of the aqueous extract by freeze-drying, changes in colors were visible but pink-red powders were obtained. In other studies, in which oven and vacuum drying were carried out at high drying temperatures, significant changes in color values were observed in tulip petals and poppy seeds [30]. Compared to natural drying, microwave drying, hot air drying, and radiant drying, freeze-drying had a positive effect in protecting the color of *Bletilla striata* flowers, and the degree of browning was low [31].

Similarly, in another study, no significant change in the color of dragon fruit was observed after freeze-drying [32]. These findings suggest that freeze-drying retains pigments to a large extent [33].

In addition to color, the yield of the microencapsulation process was determined in this study (Table 1). It was observed that the yield of RP microencapsulated with a blend of maltodextrin and guar gum or lecithin was on a comparable level, ranging from 220 to 240%, as achieved in the previous study on blue powder. The greater addition of guar gum was associated with increased efficiency, similar to that observed in the previous study [26]. Other works have only used maltodextrin for microencapsulation of extracts by lyophilization [34,35]. Moreover, in previous studies that used guar gum as a stabilizer, favorable changes were evident in the quality of the powder obtained by lyophilization [36,37]. A study also showed improvement in the thermal stability of anthocyanins lyophilized with gum Arabic [38].

### 2.2. Mineral Composition

The mineral composition of RP microencapsulated from blue cornflower powders is shown in Table 2. It was observed that the petals (C) had significantly higher mineral content than RP obtained from the aqueous extract (R2G, R4G, R2L).

It has been shown that significantly more minerals were detected in whole plants than in the extracts obtained from them [39]. As indicated by the previous study [26] as well as this study, cornflower petals are a rich source of potassium (16,411 mg/kg) and calcium (3264 mg/kg), and also contain high amounts of magnesium (1421 mg/kg) and iron (193 mg/kg). RP prepared in this study had a higher content of potassium (5240–6580 mg/kg), calcium (499–712 mg/kg), and magnesium (322–778 mg/kg) than other minerals, which were comparatively higher in blue powders [26]. The content of copper and manganese was also significantly higher in RP compared to blue powders. Whole plants usually contain more minerals than extracts from them, especially when extraction is carried out at high dilution with water. The type of solvent is also important, as in our case, the use of citric acid influenced the differentiation of the mineral content in the powders.

### 2.3. Spectral Characteristics of Microencapsulated Cornflower RP (FT-IR, FT-Raman)

**Figure 1:**
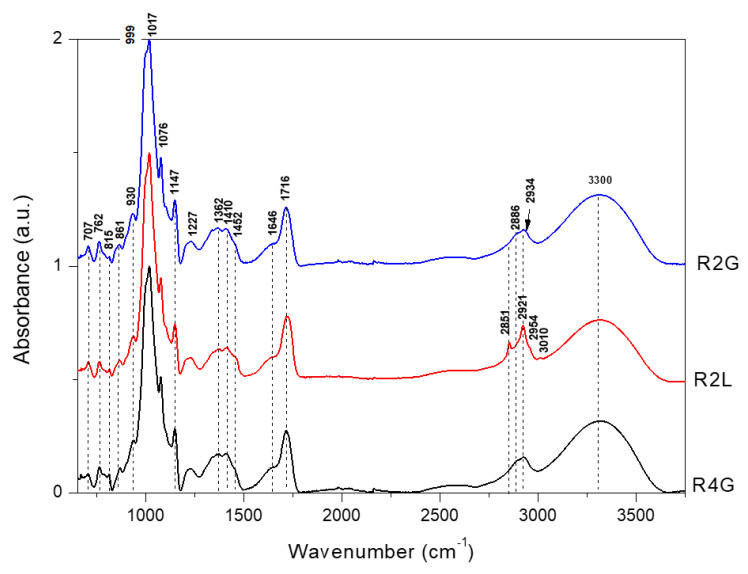
FTIR in range 650–3750 cm^−1^ for microencapsulated cornflower red powders blend with guar gum, maltodextrin, and lecithin. R2G—microencapsulated red powder from cornflower extract with 2% guar gum and 8% maltodextrin, R4G—microencapsulated red powder from cornflower extract with 4% guar gum and 6% maltodextrin, R2L—microencapsulated red powder from cornflower extract with 2% lecithin, 2% guar gum, and 6% maltodextrin.

**Figure 2:**
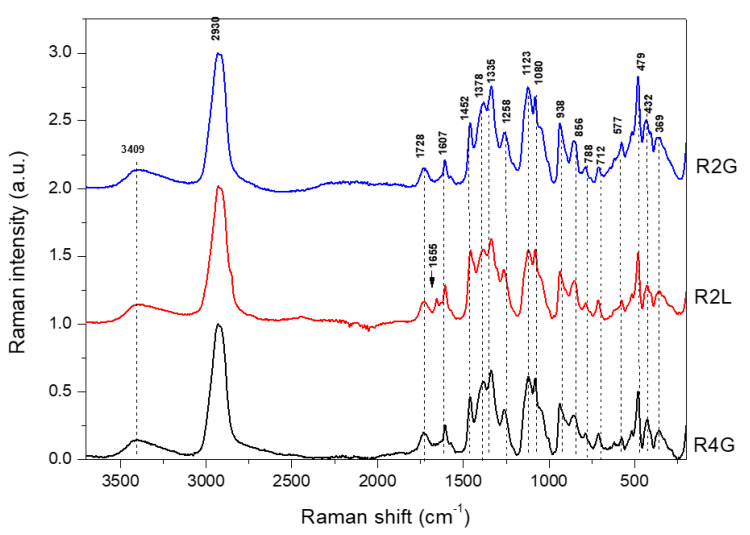
Raman spectra in range 3750–200 cm^−1^ for microencapsulated cornflower red powders blend with guar gum, maltodextrin, and lecithin. R2G—microencapsulated red powder from cornflower extract with 2% guar gum and 8% maltodextrin, R4G—microencapsulated red powder from cornflower extract with 4% guar gum and 6% maltodextrin, R2L—microencapsulated red powder from cornflower extract with 2% lecithin, 2% guar gum, and 6% maltodextrin.

The FT-IR and FT-Raman spectra of microencapsulated RP stabilized with guar gum, lecithin, and maltodextrin in the full range are presented in Figure 1 and Figure 2. In the previous study, analysis of the spectral characteristics of blue powders stabilized with guar gum, lecithin, and maltodextrin revealed that the FT-IR and FT-Raman band related to guar gum, lecithin, and maltodextrin were found to dominate over those characteristic of anthocyanins present in the powders made from cornflower petals [26]. Similarly, in the present study, the FT-IR and FT-Raman bands related to stabilizers were found to dominate over the bands characteristic of anthocyanins contained in RP extracted from cornflower petals.

RP were obtained by adding citric acid to the extract and reducing the pH from 4.7 (blue powder) to 2.2 (RP). The addition of citric acid seemed to interfere with the spectrum (FT-IR and FT-Raman spectra of citric acid are presented in Figure S1 of the Supplementary Material). In the FT-IR spectra of R2G, R4G, and R2L, the band of 1716 cm^−1^ was observed, which was comparable with that of the blue powders examined in the previous study (Figure S2a–c, Supplementary Materials) [26], while in the FT-Raman spectra, this band was observed at 1730 cm^−1^ (Figure S2d–f, Supplementary Materials).

Under different pH, anthocyanins change their color by changing the structure: benzopyrylium ring undergoes methoxylation or hydroxylation, and sugar residue undergoes acetylation. Above neutral pH, these pigments can exist in chalcone (yellow), quinoidal (blue), or hemiketal form (colorless), while at low pH, they are in the form of flavylium cation (red color). At pH 6–8, the most probable form is quinoidal [40].

Changes were the most visible in the FT-Raman spectra and less visible in the FT-IR spectra (Figure S2, Supplementary Materials). FT-IR spectra (Figure 1) of RP differed in the region 1200–1100 cm^−1^. The band at 1227 cm^−1^ was observed for red mixtures, while for blue forms [26], this band was shifted to 1240 cm^−1^ (Figure S2a–c, Supplementary Materials). These bands are probably connected with the stretching vibration of C–O in carbohydrates.

In the FT-Raman spectra (Figure 2), there were significant differences in several bands obtained for red in this study and blue forms obtained in the previous study [26]. The main difference was related to the shift of the band at 1511 cm^−1^ (blue forms) to 1573 cm^−1^ (very weak, red forms) (Figure S2d–f, Supplementary Materials). The band at 1511 cm^−1^ was attributed to phenyl ring stretching vibration and shifted to 1573 cm^−1^ under pH 4.52. In a previous study, appearance of the band at 1570 cm^−1^ was related to the effect of changes in the glucosylation of the phenyl ring [41]. Burns et al. [42] showed that the band at 1520 cm^−1^ increased in intensity, due to the delocalization of C=C in the quinoidal base at a higher pH. In addition, as the pH increased, an obvious change was observed in the absorption band at 1573 cm^−1^, which was associated with the flavylium cation of the anthocyanin [42]. Another difference was related to the shift from 734 cm^−1^ (blue form) [26] to 760 cm^−1^ (red forms) (Figure S2d–f, Supplementary Materials). In blue forms obtained in previous study, this band was attributed to aromatic ring vibration in anthocyanins. The region 640–750 cm^−1^ was probably associated with glycosylated benzopyrylium at C(5) and dependent on the nature of the sugar [41]. The band at 1607 cm^−1^ was assigned to phenyl ring vibration in anthocyanins [43]. This band did not undergo change after pH reduction, as observed in the case of pelargonidin-3-glucoside, or it disappeared at low pH, as found for cyanidin-3-glucoside and delphinidin-3-glucoside [43].

### 2.4. SEM Microstructure of Microencapsulated Cornflower RP

**Figure 3:**
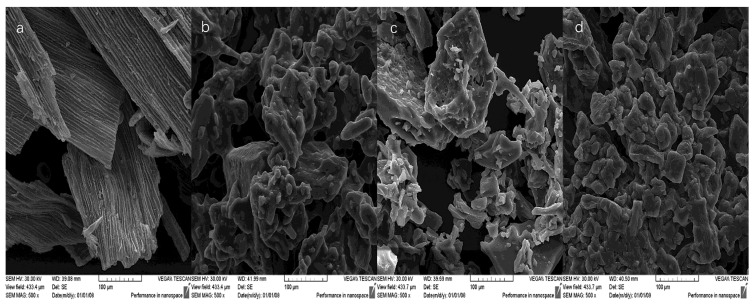
Microstructure (SEM) of cornflower petals (C) and microencapsulated red powders (R2G, R4G, R2L) obtained from cornflower extract (**a**) cornflower petals (C), (**b**) red powder from cornflower extract with 2% guar gum and 8% maltodextrin (R2G), (**c**) red powder from cornflower extract with 4% guar gum and 6% maltodextrin (R4G), (**d**) red powder from cornflower extract with 2% lecithin, 2% guar gum, and 6% maltodextrin (R2L).

The SEM microstructure of cornflower petals and the obtained RP differed significantly (Figure 3). In the images of dried cornflower petals, longitudinal stripes with flat epidermal cells were visible, as shown in the previous work. According to the literature, the epidermal cells of various flower petals can have flat shapes [44].

RP with a greater proportion of guar gum had a more amorphous structure, which is in line with the relationship observed in the previous work [26]. Drug production studies using guar gum have also reported amorphous shapes [45]. The addition of lecithin caused greater agglomeration of particles, but the resulting structure was acceptable.

### 2.5. Total Phenolic and Flavonoid Content and Antioxidant Activity of Microencapsulated Cornflower RP

Cornflower petals (C) were characterized by high TPC of 43.4 mg GAE/g DW. The powders obtained from them (R2G, R4G, R2L) also had high TPC ranging from 20.8 to 23.4 mg GAE/g DW (Table 3).

The highest phenolic content (23.4 mg GAE/g DW) was achieved when lecithin was used as the additive to red extract. In the previous study [26], similar values were obtained (19.5–26.6 mg GAE/g DW) for microencapsulated blue powders made from cornflower petals. A study showed that pomegranate seed powder had TPC of 4.9 mg GAE/g DW [46]. In a study conducted in Japan, TPC of 13 edible flowers used as food ingredients ranged from 1.47 to 13.08 mg GAE/g FW [47]. Another study showed that TPC of encapsulated roselle powder ranged between 32 and 38 mg GAE/g [48].

In cornflower petals (C), TFC was estimated at 16.8 mg QE/g, while in microencapsulated RP, it was significantly lower and ranged between 5.0 and 5.2 mg QE/g. In our previous study [26], blue powder was characterized by significantly higher TFC values, ranging from 6.0 to 6.4 mg QE/g.

The antioxidant activity of the obtained microencapsulated RP measured by ABTS, OH^•^, and RED assays ranged from 13.8 to 20.2 EC_50_ mg DW/mL, 1.9 to 2.6 EC_50_ mg DW/mL, and 31.0 to 38.7 EC_50_ mg DW/mL, respectively. Addition of lecithin resulted in higher antioxidant activity (ABTS, RED) of RP. In our previous study [26], the ability to quench ABTS radicals, as measured by the EC_50_ value of powder, was found to be significantly lower for blue powders in comparison with RP assessed in the present study. OH^•^ radical scavenging activity was higher in RP compared with blue powders [26], while antioxidant activity measured by RED assay was higher in blue powders.

## 3. Materials and Methods

### 3.1. Chemicals

Basic chemicals used in the study, ABTS (2,2-azino-bis(3-ethylbenzothiazoline-6-sulphonic acid), Folin–Ciocalteu reagent, potassium ferricyanide, FeSO_4_, sodium salicylate, ferric chloride, trichloroacetic acid, and hydrogen peroxide were purchased from Sigma-Aldrich Company (Poznan, Poland).

### 3.2. Preparation of Red Extracts of Cornflower Petals

The raw material used for preparing the extracts was organic dried blue cornflower petals (Runo, Hajnówka, Poland). Extraction (4 °C, 24 h) of cornflower petals in water (1:20), which was carried out as described in the previous study [26] resulted in a blue-colored extract. By reducing the pH value of this colored extract from 4.7 (blue) to 2.2 (red) using citric acid, a red-colored extract was obtained.

### 3.3. Preparation of Microencapsulated Cornflower RP

Red-colored powders were obtained as described in the previous work [26]. For this purpose, the extracts were microencapsulated by lyophilization with the addition of 10% stabilizers. Optimal mixtures of stabilizers, i.e., maltodextrin with guar gum and maltodextrin with lecithin, were used for microencapsulation. The samples were marked as follows: R2G (2% guar gum and 8% maltodextrin), R4G (4% guar gum and 6% maltodextrin), and R2L (2% lecithin, 2% guar gum, and 6% maltodextrin).

The mixtures containing red extract and stabilizers were frozen at −30 °C for 48 h. Freeze-drying was carried out at a temperature of 20 °C under a pressure of 63 Pa (ALPHA 1-4 lyophilizer), as described in previous studies [26,49,50]. After drying, the samples with 3% moisture content were ground into fine (particles smaller than 200 µm) powders.

### 3.4. Color Measurements of Microencapsulated Cornflower RP

Color measurements of the microencapsulated powders were obtained using a colorimeter (4Wave CR30-16; Planeta, Tychy, Poland) on the CIE-L*a*b* uniform color space [51,52]. In the CIE-L*a*b* system, the L* parameter denotes the lightness level, the a* parameter extends from green (−) to red (+), and the b* parameter extends from blueness (−) to yellowness (+). In addition, the parameter C* (chroma) denotes the color intensity and h^o^ the hue angle [7].

### 3.5. Trace Element Evaluation in Microencapsulated Cornflower RP

Before the measurements, the samples were mineralized (Mars Xpress, CEM), and the levels of potassium, zinc, copper, iron, calcium, and manganese (EN 14084:2003) [53], as well as sodium and magnesium (EN 15505:2008) [54] were determined. Flame atomic absorption spectrometry (SpektrAA 280 FS, autosampler SPS-3, thinner SIPS, Varian, Crawley, UK) was performed for estimating the content of trace elements in powders.

### 3.6. Spectral Characteristics (FT-IR, FT-Raman) of Microencapsulated Cornflower RP

Fourier transform infrared spectroscopy (FT-IR) and Fourier transform Raman spectroscopy (FT-Raman) evaluation of microencapsulated cornflower RP were performed as described previously [26].

FT-IR spectra were acquired in the range of 4000–650 cm^−1^ (Nicolet 6700 FT-IR, Thermo Scientific, Madison, WI, USA) using a Smart iTR ATR sampling accessory. FT-Raman spectra (NXR FT Raman) were acquired on a Nicolet FT-IR stand with an InGaAs detector and a CaF2 beam splitter (Thermo Scientific, Madison, WI, USA). Before obtaining the spectra, the samples were irradiated with an Nd: YAG excitation laser operating at 1064 nm. The spectra were recorded in the range of 3500–150 cm^−1^. Graphical presentation of spectra was performed using Origin Software Lab v8.5 Pro (Northampton, MA, USA).

### 3.7. Structural Characteristics of Microencapsulated Cornflower RP

The microstructure of the powders was evaluated by scanning electron microscopy (SEM) (VEGA LMU microscope, Tescan, Warrendale, PA, USA). Prior to the analysis, the samples were mounted on carbon discs using a silver tape and sprayed with gold (vacuum sublimator K-550×, Emitech, RC, Ashford, England, UK). The microscope was operated at a voltage of 30 kV, and microstructure images were taken at different magnifications (×100 and ×400).

### 3.8. Total Phenols and Flavonoid Evaluation

Extracts (methanol:water, 1:1, *v*/*v*) were first prepared for determining the content of total phenols, flavonoids, and antioxidants as previously described [26]. Total phenolic content (TPC) was determined using the Folin–Ciocalteu method with slight modifications [55], Briefly, 0.5 mL of the sample, 0.5 mL H_2_O, two ml Folin–Ciocalteu reagent (1:5 H_2_O) were added, and after 3 min, 10 mL of 10% Na_2_CO_3_, and the contents were mixed and allowed to stand for 30 min. Absorbance at 725 nm was measured in a UV–Vis spectrophotometer. The amount of total phenolics was calculated and the results were expressed in mg GAE (gallic acid equivalent) in mg·g^−1^ of dry weight (DW).

The total flavonoids content (TFC) was determined using the method described by Lamaison and Carnart [56] and by Świeca et al. [57]. Briefly, 1 mL of extract was mixed with 1 mL of 2% AlCl_3_ × 6H_2_O solution and incubated at room temperature for 10 min. Afterwards, the absorbance at 430 nm was measured. Total flavonoids content was calculated as a QE (quercetin equivalent) and expressed in mg·g^−1^ of dry weight (DW).

### 3.9. Antioxidant Activity Measurements

Antioxidant activity analyses (ABTS, reducing antioxidant power (RED), OH^•^) were performed on a plate spectrophotometer (BioTek, Model Epoch2TC, S/N 15120115). The ABTS radical scavenging activity was analyzed as described by Re et al. [58], ABTS+^•^ radicals were generated by the oxidation of ABTS with potassium persulfate. The ABTS radical cation was obtained by reacting 7 mmol/L stock solution of ABTS with 2.45 mmol/L potassium persulphate. The ABTS+^•^ solution was diluted with distilled water to an absorbance of 0.7 ± 0.05 at 734 nm. Then, 40 microliters of samples were added to 1.8 mL of ABTS+^•^ solution and the absorbance was measured at the end time of 5 min. The ability of the extracts to quench the ABTS free radical was determined using the following equation:

(1) $$\mathrm{Scavenging}\;\mathrm{activity}\;=\frac{A_{c}-A_{s}}{A_{c}}*100\;\%,$$

where A_c_ is the absorbance of the control, A_s_ is the absorbance of the sample

Reducing power (RED) was determined as described by Oyaizu [59]. First, 2.5 mL of extracts were mixed with 2.5 mL of 200 mmol/L phosphate buffer (pH 6.6) and 2.5 mL of 1% aqueous solution of potassium ferricyanide K_3_[Fe(CN_6_)]. The mixtures were incubated at 50 °C for 20 min. At the end of the incubation, 0.5 mL of 10% trichloroacetic acid was added to the mixture and centrifuged at 25× *g* for 10 min. The upper layer of solution (2.5 mL) was mixed with 2.5 mL of deionized water and 0.5 mL of 0.1% ferric chloride. The colored solution was read at 700 nm.

OH^•^ scavenging activity was determined as described by Su et al. [60]. Hydroxyl radicals were generated by the Fenton reaction in the system of FeSO_4_ and H_2_O_2_. The reaction mixture contained 0.5 mL of FeSO_4_ (8 mM), 0.8 mL of H_2_O_2_ (6 mM), 0.5 mL distilled water, 1.0 mL of extract, and 0.2 mL of sodium salicylate (20 mM). The mixture (3.0 mL) was incubated at 37 °C for 1 h. The intensity of the purple color formed was measured at 562 nm against a reagent blank and the scavenging activity was calculated:

(2) $$\mathrm{Scavenging}\;\mathrm{activity}\;\%=\left(1-\frac{A_{1}-A_{2}}{A_{0}}\right)*100\%,$$

where A_1_ is the absorbance of the control, A_1_ is the absorbance of the extract addition and A_2_ is the absorbance without sodium salicylate.

Reducing power was determined as EC_50_—the effective concentration at which the absorbance was 0.5. The antiradical activities were expressed in EC_50_ index (half maximal inhibitory concentration) which refers to the half-maximal effective concentration of antioxidants that causes a 50% decrease in activity.

### 3.10. Statistical Analyses

Statistical analyses were performed in Statistica 12.0 program (StatSoft, Krakow, Poland). All measurements were made in triplicate and means and standard deviations were calculated. Each parameter was assessed using one-way analysis of variance (ANOVA). If significant differences were observed in ANOVA at a significance level of α = 0.05, the means were compared using Tukey’s range test.

## 4. Conclusions

The results of this study showed that it is possible to obtain a deep red-colored powder using an aqueous extract of blue cornflower petals after reducing the pH of the extract from 4.7 (blue) to 2.2 (red). The extract was microencapsulated by lyophilization with the addition of 10% stabilizers (8% or 6% maltodextrin and 2% or 4% guar gum or 6% maltodextrin, 2% guar gum, and 2% lecithin). RP had the highest content of potassium, calcium, and magnesium. Analysis of spectral characteristics of the blue powders stabilized with guar gum, lecithin, and maltodextrin showed that the FT-IR and FT-Raman band related to guar gum, lecithin, and maltodextrin dominated over those characteristic of anthocyanins present in the cornflower petals. Microencapsulated red powders from cornflower blue petals are a good source of phenolic acids, they are also characterized by high antioxidant activity, especially measured by OH^•^ radical scavenging activity. Addition of lecithin contributed to higher antioxidant activity (ABTS, RED) of RP. The developed microencapsulated cornflower RP can be valuable additives to food such as sweets, jellies, puddings, drinks, or dietary supplements. Such additives have health benefits and may have a coloring function.

## Figures and Tables

**Table 1:** Color parameters of raw material and yield of powdered samples of cornflower petals.

Kind of Sample	Color Parameters					Yield of Powder (%)
	L*	a*	b*	C*	h^o^	
C	32.39 ± 1.321 ^e^	4.21 ± 0.31 ^c^	−7.98 ± 0.24 ^d^	8.89 ± 0.36 ^f^	297.78 ± 3.54 ^c^	-
R	23.90 ± 0.45 ^f^	3.79 ± 0.11 ^cd^	−1.78 ± 0.03 ^h^	4.21 ± 0.05 ^g^	328.04 ± 1.91 ^b^	-
R2G	60.41 ± 0.21 ^b^	29.70 ± 0.22 ^a^	−2.11 ± 0.08 ^fg^	29.77 ± 0.23 ^a^	355.05 ± 7.20 ^a^	224.9 ± 4.32 ^b^
R4G	59.26 ± 0.36 ^bc^	30.21 ± 0.45 ^a^	−2.74 ± 0.04 ^e^	30.28 ± 0.12 ^a^	355.25 ± 5.64 ^a^	241.8 ± 4.34 ^a^
R2L	64.76 ± 0.06 ^a^	26.79 ± 0.18 ^b^	−1.94 ± 0.05 ^g^	26.86 ± 0.18 ^b^	355.45 ± 6.49 ^a^	221.0 ± 2.28 ^b^

C—dried cornflower petals, R—red acidified extract, R2G—red powder from acidified extract (R) with 2% guar gum and 8% maltodextrin, R4G—red powder from acidified extract (R) with 4% guar gum and 6% maltodextrin, R2L—red powder from acidified (R) extract with 2% lecithin, 2% guar gum and 6% maltodextrin. Mean values ± standard deviations and the values marked with different letters are significantly different at the level α = 0.05.

**Table 2:** Mineral composition of microencapsulated cornflower red powders.

Mineral Composition								
Kind of Sample	Ca (mg/kg)	K (mg/kg)	Mg (mg/kg)	Na (mg/kg)	Fe (mg/kg)	Zn (mg/kg)	Cu (mg/kg)	Mn (mg/kg)
C	3264 ± 101 ^a^	16411 ± 457 ^a^	1421 ± 84 ^a^	79.7 ± 2.9 ^c^	193 ± 10.1 ^a^	44.6 ± 2.3 ^a^	8.31 ± 0.41 ^a^	59.9 ± 2.31 ^a^
R2G	499 ± 19 ^c^	5240 ± 187 ^c^	322 ± 25 ^d^	381 ± 21 ^a^	34.9 ± 1.4 ^cd^	7.8 ± 0.4 ^b^	3.95 ± 0.13 ^b^	7.916 ± 0.32 ^bc^
R4G	545 ± 27 ^c^	5480 ± 234 ^c^	394 ± 24 ^c^	334 ± 23 ^b^	39.4 ± 2.5 ^b^	7.6 ± 0.3 ^bc^	4.32 ± 0.20 ^b^	8.39 ± 0.27 ^bc^
R2L	712 ± 55 ^b^	6580 ± 321 ^b^	778 ± 32 ^b^	327 ± 19 ^ab^	41.6 ± 1.7 ^c^	8.9 ± 0.4 ^d^	3.64 ± 0.12 ^c^	8.98 ± 0.45 ^b^

C—cornflower petals, R2G—microencapsulated red powder from cornflower extract with 2% guar gum and 8% maltodextrin, R4G—microencapsulated red powder from cornflower extract with 4% guar gum and 6% maltodextrin, R2L—microencapsulated red powder from cornflower extract with 2% lecithin, 2% guar gum and 6% maltodextrin. Mean values ± standard deviations and the values in the same column marked with different letters are significantly different at the level α = 0.05.

**Table 3:** Total phenolic and flavonoid content and antioxidant activity of microencapsulated red powders obtained from cornflower petals.

Kind of Sample	TPC (mgGAE/g DW)	TFC (mg QE/g)	ABTS EC_50_ (mgDW/mL)	OH^•^ EC_50_ (mgDW/mL)	RED EC_50_ (mgDW/mL)
C	43.39 ± 1.43 ^a^	16.81 ± 0.57 ^a^	7.53 ± 0.38 ^c^	7.15 ± 0.24 ^a^	10.88 ± 0.39 ^c^
R2G	21.63 ± 1.01 ^c^	5.15 ± 0.20 ^b^	20.20 ± 0.44 ^a^	2.24 ± 0.09 ^c^	38.74 ± 1.78 ^a^
R4G	20.76 ± 1.01 ^c^	5.23 ± 0.23 ^b^	19.81 ± 0.56 ^a^	1.91 ± 0.04 ^d^	36.51 ± 0.97 ^a^
R2L	23.42 ± 0.71 ^b^	5.009 ± 0.16 ^b^	13.84 ± 0.64 ^b^	2.61 ± 0.09 ^b^	31.05 ± 1.18 ^b^

C—cornflower petals, R2G—microencapsulated red powder from cornflower extract with 2% guar gum and 8% maltodextrin, R4G—microencapsulated red powder from cornflower extract with 4% guar gum and 6% maltodextrin, R2L—microencapsulated red powder from cornflower extract with 2% lecithin, 2% guar gum and 6% maltodextrin. Mean values ± standard deviations and the values in the same column marked with different letters are significantly different at the level α = 0.05.

## Data Availability

The data presented in this study are available on request from the corresponding authors.

## Supplementary Materials

The following supporting information can be downloaded at: https://www.mdpi.com/article/10.3390/molecules27103094/s1. Figure S1: SM. FT-IR (a) and FT-Raman (b) spectra of citric acid. The most characteristic bands that could influence the spectra of samples are marked; Figure S2: SM. FT-IR (a–c) and FT-Raman (d–f) spectra of blue and red powders blend with maltodextrin (M), guar gum (G) and lecithin. FT-IR bands that differ between red and blue samples are marked with navy blue arrow. Raman bands characteristic for anthocyanins are marked with green arrows, additionally with shading the region were changes in anthocyanins bands were denoted. R2G/B2G-microencapsulated red (R)/blue (B) powder from cornflower extract with 2% guar gum and 8% maltodextrin, R4G/B4G—microencapsulated red (R)/blue (B) powder from cornflower extract with 4% guar gum and 6% maltodextrin, R2L/B2L—microencapsulated red (R)/blue (B) powder from cornflower extract with 2% lecithin, 2% guar gum and 6% maltodextrin.
