# Supplementary material for: Microencapsulated Red Powders from Cornflower Extract—Spectral (FT-IR and FT-Raman) and Antioxidant Characteristics
Source: Molecules. 2022 May 11;27(10):3094. doi: 10.3390/molecules27103094 (PMC9147898; doi:10.3390/molecules27103094)
Supplement: Supplementary file 1 [file molecules-27-03094-s001.zip › molecules-1689318-supplementary.pdf]

molecules

Article

Microencapsulated Red Powders from Cornflower Extract—Spectral (FT-IR and FT-Raman) and Antioxidant Characteristics

Renata Różyło [1,]* Monika Szymańska-Chargot [2,]*, Artur Zdunek [2], Urszula Gawlik-Dziki [3] and Dariusz Dziki [4]

SUPPLEMENTARY MATERIAL

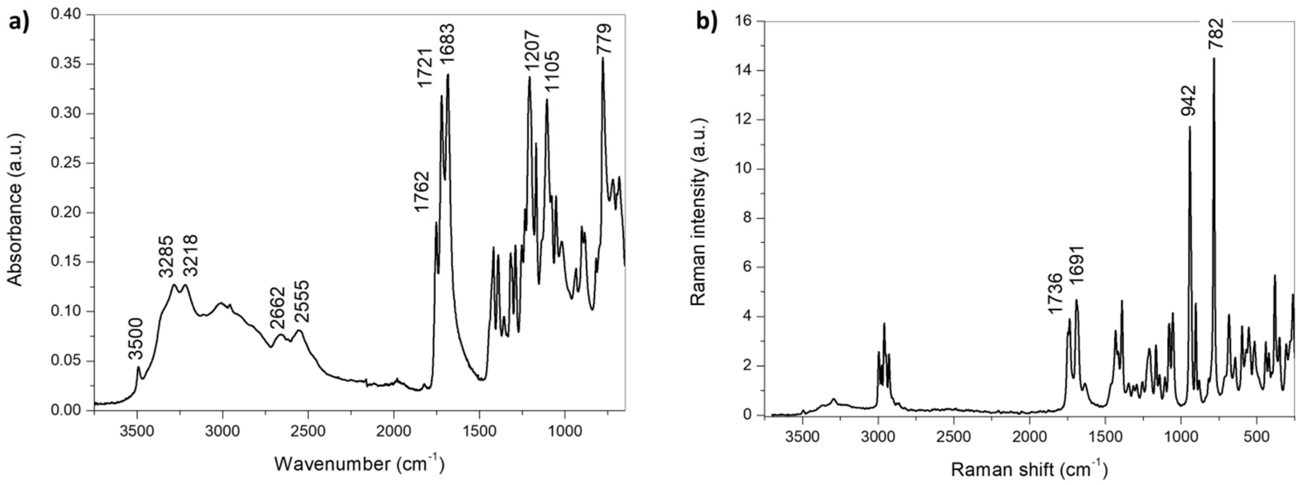

Figure S1. FT-IR (a) and FT-Raman (**b**) spectra of citric acid. The most characteristic bands that could influence the spectra of samples are marked.

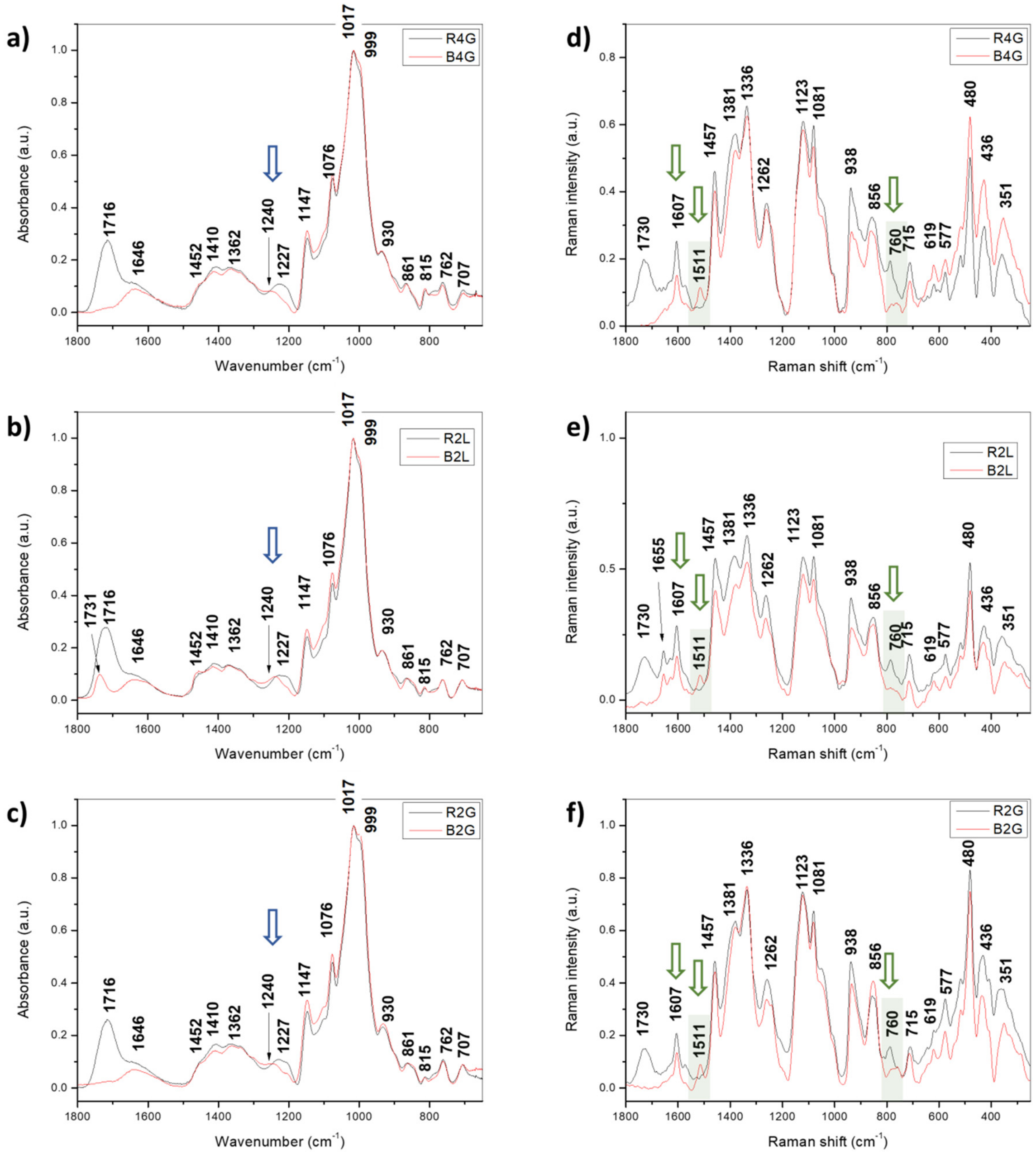

Figure S2. FT-IR (**a**–**c**) and FT-Raman (**d**–**f**) spectra of blue and red powders blend with maltodextrin (M), guar gum (G) and lecithin. FT-IR bands that differ between red and blue samples are marked with navy blue arrow. Raman bands characteristic for anthocyanins are marked with green arrows, additionally with shading the region were changes in anthocyanins bands were denoted. R2G/B2G- microencapsulated red (R)/blue (B) powder from cornflower extract with 2% guar gum and 8% maltodextrin, R4G/B4G – microencapsulated red (R)/blue (B) powder from cornflower extract with 4% guar gum and 6% maltodextrin, R2L/B2L – microencapsulated red (R)/blue (B) powder from cornflower extract with 2% lecithin, 2% guar gum and 6% maltodextrin.
